# Extending the CASO-N24 to Late Adolescence: Psychometric Properties and Measurement Equivalence in a Peruvian School Sample

**DOI:** 10.3390/healthcare14081029

**Published:** 2026-04-14

**Authors:** Haydee Mercedes Aguilar-Armas, Velia Graciela Vera-Calmet, Marco Agustín Arbulú Ballesteros, Lucy Angélica Yglesias-Alva, Hugo Martin Noé Grijalva, Milagros del Carmen Quispe Villarreal

**Affiliations:** 1 Institute for Research in Science and Technology, Chepén Campus, César Vallejo University, Trujillo 13001, Peru; haguilarar@ucvvirtual.edu.pe (H.M.A.-A.); vverac@ucv.edu.pe (V.G.V.-C.); hnoe@ucv.edu.pe (H.M.N.G.); mquispevi@ucv.edu.pe (M.d.C.Q.V.); 2 Departamento de Estadística, Facultad de Ciencia Físicas y Matemáticas, Universidad Nacional de Trujillo, Trujillo 13001, Peru; lyglesias@unitru.edu.pe

**Keywords:** anxiety, adolescents, mental health, psychological assessment, psychometrics

## Abstract

**Highlights:**

**What are the main findings?**

- The six-factor multidimensional structure of the CASO-N24 was replicated in 710 Peruvian adolescents aged 12 to 17 years through exploratory and confirmatory factor analyses with satisfactory goodness-of-fit indices.
- Complete measurement invariance by age group (12–15 vs. 16–17 years) was achieved at configural, metric, scalar, and residual levels, supporting the age extension of the instrument beyond its originally validated range.
- Partial invariance by gender was found, with differential item functioning identified in item 17, indicating response specificities associated with gender in the interaction with the opposite sex dimension.

**What are the implications of the main findings?**

- Convergent validity was supported by moderate-to-high correlations with the ASQ-14 in both males (r = 0.622) and females (r = 0.604), which consistently links social anxiety to a theoretically related measure of perceived stress in adolescents.
- Internal consistency was adequate for the total scale (ω = 0.95) and subscales (ω = 0.69–0.82), positioning the CASO-N24 as a psychometrically robust tool for early detection of social anxiety in Peruvian school settings.

**Abstract:**

**Background**: Social anxiety in adolescence is a prevalent mental health concern characterized by intense fear of negative evaluation in social situations. The Social Anxiety Questionnaire for Adolescents (CASO-N24) is a Spanish-language instrument requiring validation in Peruvian populations. **Objective**: This study aimed to validate the CASO-N24 in Peruvian adolescents aged 12–17 years, extending its application beyond the original 9–15-year range, and examine its psychometric properties including factorial structure, measurement invariance, nomological validity, and internal consistency. **Methods**: A stratified probability sample of 710 adolescents (352 males, 358 females; M = 14.82 years, SD = 1.45) from four northern Peruvian educational centers completed the CASO-N24 and ASQ-14. Exploratory and confirmatory factor analyses, multigroup invariance testing by age and gender, nomological validity assessment, and reliability estimation (Cronbach’s α and McDonald’s ω) were conducted using polychoric correlations and robust estimation methods. **Results**: The six-factor structure was replicated, explaining 47.13% of variance with factor loadings ranging 0.48–0.78. Model fit indices were excellent (GFI = 0.981, AGFI = 0.976, NFI = 0.971, SRMR = 0.046). Complete measurement invariance was achieved across age groups (12–15 vs. 16–17 years). Partial invariance by gender was observed, with differential item functioning identified in item 17. Nomological validity was confirmed through moderate-to-high correlations with ASQ-14 (males: r = 0.622; females: r = 0.604). Internal consistency was adequate (total scale ω = 0.95; subscales ω = 0.69–0.82). **Conclusions**: The CASO-N24 demonstrated robust psychometric properties for assessing social anxiety in Peruvian adolescents aged 12–17 years, supporting its multidimensional structure and utility for early detection in school settings while highlighting gender-specific response patterns warranting clinical consideration.

## 1. Introduction

Social anxiety in adolescence is a widespread mental health problem, characterized by intense and persistent negative emotions in social situations, with a marked apprehensive anticipation of possible evaluations by others, generating significant distress and functional impairment in daily life, affecting their emotional well-being and performance in habitual activities [1,2,3]. This stage is critical as demands for social interaction increase; high sensitivity to evaluation by peers and adults favors the appearance of social anxiety symptoms in some young people [4,5,6]. For this reason, many typical social fears tend to be transitory, but in certain cases, the fear of interaction becomes excessive, limiting normal social coexistence, triggering strong anxiety reactions and avoidance behaviors that interfere with their social development.

Rates close to 20% of adolescents with anxiety symptoms have been reported in North America and 17% in Europe [7,8], trends that may have worsened after the COVID-19 pandemic [9,10]. Social anxiety affects approximately 8% of adolescents globally [11]. In Peru, epidemiological evidence is limited; in Lima, a study with schoolchildren aged 13 to 17 years found that 25.9% were at a severe level of social anxiety [12]. Therefore, it is considered a public health problem given its high prevalence and its adverse impact on the school, social, and emotional adaptation of young people [1,3,13]. Beyond its clinical relevance, social anxiety in adolescence carries significant economic and societal implications; adolescents who develop chronic social anxiety are more likely to underperform academically, experience employment difficulties, and show increased utilization of mental health services in adulthood, thereby placing a greater burden on public health systems.

The manifestations of social anxiety can lead to persistent patterns of avoidance of interactions, difficulties in emotional regulation, and problems establishing healthy interpersonal bonds [14,15,16]. Studies have shown that adolescents tend to experience intense fear of negative evaluation and show cognitive biases toward threat perception in social situations, which significantly deteriorates their well-being and daily performance [17,18], increasing the risk of depression, social isolation, or other problems if not addressed in time. Detection and intervention are fundamental; therefore, it is necessary to have psychometrically robust assessment instruments to identify adolescents at risk before symptoms worsen or comorbidities develop [19,20].

The structured evaluation of this construct provides empirical information to guide prevention and treatment programs, particularly in the school context, where intervention strategies based on objective results can be implemented [9,10,21,22,23,24,25].

Given the need to specifically assess social anxiety in the youth population, Caballo and collaborators developed the Social Anxiety Questionnaire for adults (CASO-A30), and subsequently an adapted version for the child and adolescent population. The Social Anxiety Questionnaire for Adolescents (CASO-N24) is the adaptation of this instrument aimed at adolescents and children, originally formulated in Peninsular Spanish [26]. The CASO-N24 emerges from the adult instrument already validated in various Spanish-speaking countries [27,28], preserving the essence of evaluating fear in typical social situations of the Ibero-American context. The version for younger populations consists of 24 items and almost completely reproduces the factorial structure of the adult questionnaire, adapting some contents to the adolescent environment. Previous studies in different Latin American countries have confirmed that the CASO, in its different versions, presents solid psychometric properties, finding consistency and stability in its multifactorial structure, as well as high coefficients of internal consistency, and evidence of convergent validity with other measures of social phobia [1,2,29]. Being an instrument originally constructed in Spanish, the CASO minimizes translation biases present in scales imported from other languages and is culturally more pertinent for Spanish-speaking populations [2,29]. Although other instruments are available for assessing social anxiety in adolescents—such as the Social Anxiety Scale for Adolescents (SAS-A), the Social Phobia and Anxiety Inventory for Children (SPAI-C), and the Liebowitz Social Anxiety Scale for Children and Adolescents (LSAS-CA)—these were originally developed in English and require translation and cross-cultural adaptation processes that may introduce measurement bias when applied in Spanish-speaking populations. In contrast, the CASO-N24 was natively constructed in Spanish within an Ibero-American framework, which confers greater linguistic and cultural pertinence for the target population of this study. These characteristics have positioned the CASO-N24 as a valuable tool for clinical and psychoeducational evaluation of social anxiety in adolescents, allowing for precise case detection and guiding intervention strategies.

The CASO-N24 is based on the cognitive-behavioral model of social phobia by Clark and Wells [30] and Rapee and Heimberg [31], according to which people with social anxiety interpret social situations as potentially threatening, focus their attention on possible signs of negative evaluation, and resort to avoidance, thus maintaining the problem. Caballo [26] extended this model to the adolescent context, incorporating developmental and social learning variables specific to this stage. The CASO-N24 assumes a multidimensional nature of social anxiety, evaluating fears across six situational domains typical of adolescence. Its items cover: (1) performance in public and interaction with authority figures (e.g., speaking in class or addressing a teacher), where fear of evaluation by high-status persons prevails [32]; (2) interaction with people of the opposite sex, associated with relational insecurity and sensitivity to romantic rejection [33]; (3) contact with strangers, linked to beliefs of possible social incompetence in new contexts [34]; (4) everyday social situations (gatherings, parties, etc.), which can generate progressive avoidance in the face of possible social judgment [35]; (5) assertive expression of annoyance or disagreement, that is, the fear of confronting or setting limits due to fear of disapproval by the other; and (6) fear of being observed or ridiculed in public, considered a central core of social anxiety, often accompanied by self-referential cognitive biases [36].

Despite the widespread use of the CASO-N24 in Hispanic contexts, there is no validated adaptation for the Peruvian adolescent population to date. This absence represents an important gap, given that sociocultural differences could influence the manifestation of social anxiety and the psychometric functioning of the instrument. It is necessary to examine whether the factorial structure and properties of the CASO-N24 are replicated in Peruvian adolescents, thus ensuring construct validity in our setting. Previous studies of the CASO-N24 have focused mainly on children and adolescents in general, so its specific behavior in adolescents aged 16–17 years, an age range corresponding to late adolescence, is unknown. This stage involves particular contexts—such as the completion of the school stage and the transition to higher education or young adult life—that could nuance both social anxiety levels and the interpretation of certain questionnaire items.

The main objective of this study was to psychometrically validate the CASO-N24 in Peruvian adolescents. In particular, the following specific objectives were proposed: (1) to detect the factorial structure of the CASO-N24 through exploratory and confirmatory factor analysis, (2) to evaluate the factorial invariance of the instrument according to age group and gender, (3) to gather evidence of convergent validity by examining the correlation between the CASO-N24 and another related measure (the ASQ-14, abbreviated stress questionnaire for adolescents), and (4) to estimate the internal reliability of the instrument through Cronbach’s alpha and omega coefficients.

Based on previous findings, it was hypothesized that the CASO-N24 would present a multidimensional factorial structure consistent with Caballo’s original proposal.

## 2. Materials and Methods

### 2.1. Design

This study falls within the category of instrumental research [37] with a cross-sectional design, and in accordance with international guidelines for the rigorous adaptation and translation of psychometric instruments [38,39].

### 2.2. Participants

A stratified probability sampling was conducted in four educational centers in northern Peru, achieving a participation of 710 adolescents (males = 352; females = 358) with ages ranging from 12 to 17 years (M = 14.82, SD = 1.45).

**Figure 1:**
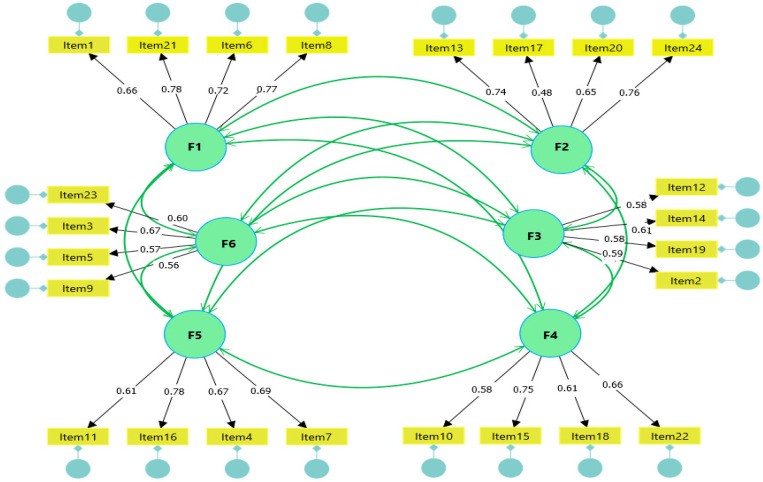
Path diagram of the confirmed factorial model with standardized loadings. Numbers on arrows represent standardized factor loadings. Green circles represent latent factors (F1–F6); rectangles represent observed items; small circles represent error terms.

The stratification was segmented by gender, academic grade, and center of origin. Prior to statistical treatment, a data cleaning phase was executed to detect inconsistencies. Protocols with monotonous response patterns or with an omission percentage greater than 5% were identified and excluded; for cases with minimal missing values, listwise deletion was applied, a procedure that guarantees the integrity and precision of correlation matrices in the presence of random losses [40]. Regarding participant flow: a total of 745 adolescents were initially approached across the four educational centers; 12 declined participation, and 23 were excluded during data cleaning (15 due to monotonous response patterns and 8 due to omission rates exceeding 5%), yielding a final analytic sample of 710 participants. A detailed participant flow diagram following STROBE guidelines is provided as Figure 1.

### 2.3. Instruments

Sociodemographic form. An ad hoc questionnaire was designed for sample characterization, which allowed the collection of information regarding age, sex, and educational level. Additionally, family environment variables were collected, such as cohabitation with both parents and number of siblings, as well as the participants’ place of origin.

The CASO-N24 instrument is designed to assess social anxiety in adolescents through 24 items distributed across six dimensions, which are answered on a 5-point Likert scale, where participants indicate the degree of anxiety they experience in each situation (from 1 = Never to 5 = Always). The subscales include: Speaking in public or interacting with adults (6 items), Interaction with people of the opposite sex (4 items), Being ridiculed (4 items), Assertive expression of annoyance (4 items), Interaction with strangers (4 items), and Acting in public (4 items). The CASO-N24 in its original version was administered to a sample of 1067 adolescents aged 9 to 15 years and yielded an internal consistency coefficient (Cronbach’s α) for the total scale of 0.93, with subscales whose values ranged between 0.88 and 0.91, evidencing excellent reliability [26]. Factor analysis revealed a six-factor structure, with factor loadings greater than 0.50 and cumulative explained variance greater than 60%, which supports the soundness of the internal structure. In terms of validity, a significant correlation was found between the total instrument score and external measures of social anxiety (r ≈ 0.62), confirming convergent validity, while the low correlation between subscales (r < 0.45) supports its discriminant validity. The original Spanish version was used, eliminating the risk of translation or cultural adaptation biases. For the present study, the age extension was performed with respect to the original model.

Convergent validity was assessed with the Adolescent Stress Questionnaire, abbreviated version (ASQ-14). Composed of 14 items, on a 5-point Likert scale ranging from 1 (“not at all stressful”) to 5 (“very stressful”). Unidimensional scale. Regarding reliability, the original study reported adequate internal consistency for the total score (α ≈ 0.85) and satisfactory temporal stability through test–retest at 4 weeks (r ≈ 0.81) [41]. Regarding validity, evidence was provided based on (a) internal structure (unidimensional model fit and invariance by age/stage) and (b) relationship with other variables, with positive associations observed with manifestations of stress, anxiety, depression, and emotional/behavioral problems, and negative associations with life satisfaction, in the theoretically expected direction for a perceived stress indicator. It is linked to the theoretical support of accumulated psychological and behavioral distress throughout life.

The substantive contribution of this study lies in the age extension of the instrument; while the original model focused on ages 9 to 15 years, this research expands its application and validation to the 12 to 17 years range. It should be noted that no linguistic modifications or cultural adaptations were made, given that the original Spanish version showed optimal intelligibility for the target population. This decision was supported by several considerations: (a) the CASO-N24 was originally constructed in Peninsular Spanish with items designed to reflect common social situations across the Ibero-American context, and its content has demonstrated adequate comprehension in multiple Latin American samples [1,2,29]; (b) prior to administration, a pilot evaluation was conducted with a subsample of 30 adolescents from the target population to verify item comprehension and response adequacy, revealing no difficulties in understanding the instructions or item content; and (c) the content validity assessment by nine expert judges, using Aiken’s V coefficient [42,43], confirmed that all items were semantically clear and culturally pertinent for the Peruvian adolescent context. Nonetheless, the absence of a formal cognitive interviewing or systematic cultural adaptation procedure is acknowledged as a methodological consideration that future studies may wish to address.

### 2.4. Procedure

The application was in-person and directed by professional psychologists during school hours, within tutoring hours, with a duration of approximately thirty minutes. Additionally, detailed and thorough information was provided about the objectives and procedures of the study, as well as specifying the potential risks and benefits. Finally, operational measures were implemented to guarantee privacy and responsible treatment of the collected data.

### 2.5. Data Analysis

Statistical treatment was executed in RStudio (v.2023.06.0) and Jamovi (v.2.4) environments. Regarding data treatment, missing values were identified in less than 5% of the sample; being minimal and random, listwise deletion was proceeded with. Due to the ordinal nature of the data and the absence of multivariate normality, confirmed by the Mardia coefficient, polychoric correlation matrices were used [44]. Previously, the absence of extreme multicollinearity was evaluated using the determinant of the correlation matrix. Likewise, content validity was determined through Aiken’s V with the judgment of nine experts [43].

To examine the internal structure, an Exploratory Factor Analysis (EFA) was initiated using the Principal Axis Factoring method with oblique Promax rotation. Factor retention was based on Horn’s Parallel Analysis, a technique that mitigates overextraction by comparing empirical eigenvalues with random simulations [45]. Item communalities, cross-loadings, and the full pattern matrix versus structure matrix are reported in Table 1 to facilitate a comprehensive evaluation of the EFA solution. Subsequently, dimensionality was ratified through a Confirmatory Factor Analysis (CFA) with the Unweighted Least Squares (ULS) estimator, reporting GFI, AGFI, NFI, RFI, and SRMR indices, excluding RMSEA due to the nature of ULS fit [46,47]. It should be noted that CFI and TLI are not directly derivable under the ULS fit function as implemented in AMOS, since these indices rely on the chi-square statistic from maximum likelihood or weighted least squares estimation; therefore, their absence is methodologically justified rather than an omission. No post hoc model modifications were performed; the hypothesized six-factor structure was tested as specified without re-specification based on modification indices, preserving confirmatory rigor. A sensitivity analysis using DWLS/WLSMV estimation is recommended and is provided as Table 2. During the CFA, residuals and modification indices were inspected to ensure model parsimony.

The stability of the instrument was evaluated through a multigroup measurement invariance analysis (sex and age), comparing configural, metric, and scalar models. Given that CFI and TLI require chi-square-based estimation, the multigroup invariance analysis was conducted using the DWLS estimator (with robust corrections), which permits the computation of these indices. For the equivalence decision, variations in the comparative fit index (ΔCFI) and Tucker–Lewis index (ΔTLI) were applied following Chen’s [48] criterion. Specifically, invariance was tested sequentially at four levels (configural, metric, scalar, and residual), with ΔCFI ≤ 0.010 and ΔTLI ≤ 0.010 as decision criteria; additionally, ΔSRMR was monitored as a supplementary index. Consistent with current methodological recommendations for ordinal data, ΔSRMR ≤ 0.030 and ΔRMSEA ≤ 0.015 were also applied as supplementary criteria in the DWLS/WLSMV sensitivity analysis (Table 2). Results are presented in a compact stepwise table. Given the finding of discrepancies in intercepts, partial invariance was explored to identify differential item functioning [49]. For the gender analysis, the partial invariance approach involved freeing the intercept of item 17 (belonging to the interaction with the opposite sex dimension), which was identified as the source of differential item functioning; all remaining item intercepts were constrained to equality. Under this partial scalar model, valid mean comparisons can be made on all latent factors except the interaction with the opposite sex dimension, where caution is warranted. Discriminant validity was verified with the Heterotrait–Monotrait ratio (HTMT < 0.85) [50]. The complete HTMT matrix is presented in Table 3, and composite reliability (CR) and average variance extracted (AVE) values per factor are provided in Table 4.

Finally, convergent and nomological validity were evaluated by analyzing the association of CASO-N24 with perceived stress (ASQ-14), previously verifying bivariate normality with the Henze–Zirkler test [52]. Internal consistency was estimated using McDonald’s Omega coefficient (ω), supplemented with 95% confidence intervals [53].

### 2.6. Ethical Considerations

The study strictly adhered to the ethical principles of the Declaration of Helsinki [54] and the guidelines for research with minors [55]. Anonymity and confidentiality were guaranteed through informed consents and assents. The research protocol was reviewed and approved by the Comité de Ética en Investigación de la Escuela de PSICOLOGÍA (approval no. N.° 01210-2024/CEI-PSI, approval date: 21 September 2024), ensuring compliance with standards of integrity and protection of participants.

## 3. Results

### 3.1. Exploratory Factor Analysis

The identification of the latent structure of the instrument was performed through an exploratory factor analysis (EFA) using the principal axis factoring method with oblique Promax rotation. Previously, the adequacy of the correlation matrix was validated with a KMO index of 0.909 and a statistically significant Bartlett’s sphericity test, χ^2^(276) = 6130.82, *p* < 0.001, confirming the appropriateness of proceeding with factor extraction [56,57]. The determinant of the matrix close to zero ratified the absence of extreme multicollinearity.

The determination of the number of dimensions to retain was based on Horn’s [58] Parallel Analysis (PA), a technique that mitigates the risk of overextraction by comparing empirical eigenvalues with those derived from random simulations [45]. The PA suggested a six-factor solution that explained 47.13% of the total cumulative variance. As detailed in Table 1, all main factor loadings ranged between 0.44 and 0.83, with no problematic cross-loadings that would compromise the parsimony of the factorial structure [59,60].

### 3.2. Confirmatory Factor Analysis

Dimensionality ratification was proceeded with through a confirmatory factor analysis (CFA). Given the ordinal nature of the items and the absence of multivariate normality, evidenced by the critical ratio value of multivariate kurtosis (29.13 > 5), indicating a significant deviation from normality (*p* < 0.01), the Unweighted Least Squares (ULS) estimator was selected, recognized for its asymptotic consistency and robustness against non-normal distributions [61,62] (Figure 1).

Factor weights (Table 2) ranged between 0.48 and 0.78, meeting the minimum criterion of 0.40 proposed by Hair et al. [51] for adequate representation of constructs. Global fit was evaluated using indices compatible with the ULS estimator in AMOS; it should be noted that RMSEA is not reported because the ULS fit function lacks a probabilistic likelihood basis, which makes the mathematical derivation of the non-centrality parameter impossible [63].

Upon verifying the findings in Table 3, the empirical adequacy of the structural solution is evident. The normed chi-square ratio (CMIN/df = 1.550) satisfies the parsimony criteria by falling well below 3.0 [47]. This support is consolidated with a GFI of 0.981 and an AGFI of 0.976; both records exceed the 0.95 threshold, indicating that the model successfully captures the variance and covariance of the sample [47,51]. Similarly, the incremental indices NFI (0.971) and RFI (0.966) validate the soundness of the proposed structure compared to independence models [47,51]. Regarding residuals, the SRMR of 0.046, below the 0.05 limit, confirms that the average discrepancy between the observed and implied matrices is marginal, lending rigor to the applied ULS estimation [47,51].

Discriminant validity of latent constructs was confirmed through the Heterotrait-Monotrait (HTMT) index. The obtained values, all below 0.85, support adequate theoretical differentiation between factors [50]. This result coincides with the interfactorial correlations shown in Table 4, which reflect appropriate levels of statistical independence between dimensions.

Correlations between the factors under study range from 0.43 to 0.69, suggesting the absence of redundant overlaps between dimensions (See Table 4).

### 3.3. Multigroup Measurement Invariance

Measurement equivalence was analyzed through a hierarchical multigroup analysis following the guidelines of Cheung and Rensvold [64] and Chen [48], where changes in the comparative fit index (ΔCFI) and Tucker–Lewis index (ΔTLI) less than or equal to 0.010 support the maintenance of invariance [65].

#### 3.3.1. Invariance by Age Group (12–15 vs. 16–17 Years)

The evaluation of measurement equivalence between age groups was performed using hierarchical models. In this sample, evidence of satisfactory metric invariance was obtained; although the chi-square difference was significant (χ^2^ = 24.65; df = 18; *p* = 0.002), the ΔIFI (0.002) and ΔTLI (0.001) values did not exceed the critical threshold of 0.010 [64]. When verifying whether the behavior of the scale was consistent across ages, the results were stable at all evaluated levels, from the configural to the residual model. This suggests that the questionnaire functions similarly in both ranges, so it was not necessary to segment the subsequent analysis by age. The stability of parameters indicates that the intersections between items remain stable across groups [66], and measurement errors do not vary significantly between developmental stages (Table 5 and Table 6).

#### 3.3.2. Invariance by Gender

In this sample, the questionnaire demonstrated satisfactory equivalence between groups both in its factorial structure and in the strength of the relationship between items and their latent dimensions, corresponding to configural and metric levels. Although the chi-square difference between models was significant, χ^2^ = 99.58, df = 18, *p* < 0.001, the ΔIFI (0.009) and ΔTLI (0.008) values fell within acceptable tolerance ranges [48,64].

However, when evaluating scalar invariance compared to the metric model, the ΔIFI (0.059) and ΔTLI (0.062) values exceeded the established thresholds (Table 7 and Table 8). Manual inspection of intercepts revealed that item 17 of the questionnaire presented the greatest discrepancy; the identified difference for this item corresponds to the absolute numerical discrepancy between the unstandardized intercepts (τ) estimated for males and females, suggesting the presence of differential item functioning (DIF) by gender [49]. A similar pattern was observed when evaluating residual invariance, where differences were also notably significant.

This finding allows documenting the response specificity associated with that symptom as a substantive research result, validating that, although direct comparison of means between males and females should be performed under a partial invariance approach, the structure of the instrument remains stable between the evaluated groups [67,68,69].

### 3.4. Comparison of Factor Loadings and Factorial Correlations by Gender

To detect possible divergences between males and females, factor loadings and interdimensional correlations were reviewed. This analysis was feasible because the multigroup analysis demonstrated that the relationships between items and their respective dimensions remain consistent in both genders, meeting the metric invariance criterion.

**Figure 2:**
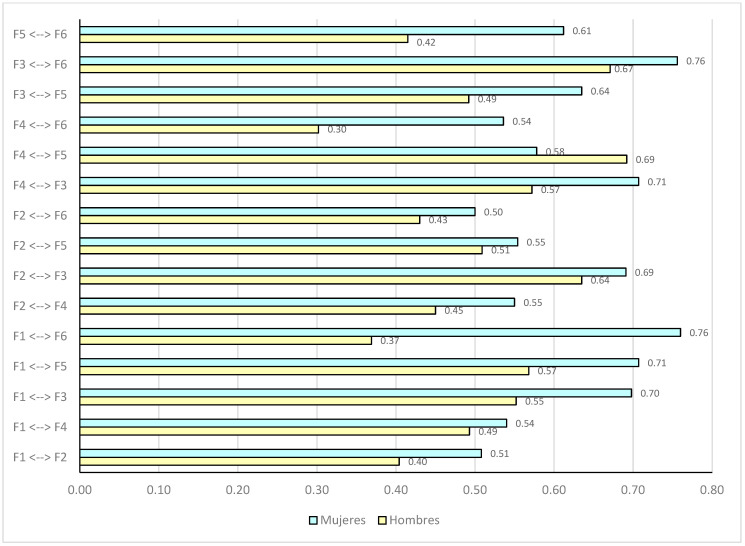
Comparison of correlations between dimensions of the anxiety questionnaire.

As shown in Table 9, although some factor loadings present slightly higher values in the female group, the items are associated with their respective dimensions analogously in both groups. This stability in the relationship pattern supports the structural consistency of the model by gender. Likewise, Figure 2 presents correlations by gender, showing direct associations in both groups. In the case of males, relationships of greater magnitude are observed between F1 ↔ F6 (r = 0.76) and F3 ↔ F6 (r = 0.76), while in females some associations, such as F2 ↔ F6 (r = 0.50), show lower values. This explains that the strength of relationships between dimensions varies by gender.

### 3.5. Content Validity

Nine experts evaluated content adequacy. Consensus was quantified using Aiken’s V [70], obtaining a general average of 0.930 (95% CI [0.715, 0.998]), with specific values for clarity (0.924; 95% CI [0.709, 0.996]), coherence (0.935; 95% CI [0.723, 1.000]), and relevance (0.929; 95% CI [0.715, 0.998]). Additionally, 95% confidence intervals (CI) were estimated using the score method [71], whose lower limits were above 0.70, which ratifies content validity [43].

### 3.6. Convergent Validity and Nomological Validity

As a preliminary step to the association analysis, the assumption of bivariate normality between the total scores of the CASO-N24 and ASQ-14 was evaluated using the Henze–Zirkler (HZ) test. Results showed adequate fit to the multivariate normal distribution in both the male group (HZ = 0.54, *p* = 0.915) and the female group (HZ = 0.66, *p* = 0.487). This finding technically justified the use of Pearson’s correlation coefficient (r) to evaluate convergence between constructs [52].

The analysis revealed a direct and statistically significant relationship in both males (r = 0.622, *p* < 0.001) and females (r = 0.604, *p* < 0.001). According to Blanca et al. [41] and Byrne et al. [72], these magnitudes ratify convergent validity, by linking coherently with a theoretically related measure of adolescent stress [73].

### 3.7. Internal Consistency of the Instrument

Internal consistency was determined using McDonald’s omega (ω) coefficient and Cronbach’s alpha (α). Following current recommendations [53,74], ω was reported as the main estimator, accompanied by its 95% confidence interval, calculated using the bootstrap method [75]. Global reliability (ω = 0.95) indicates excellent internal consistency [76,77]. Additionally, composite reliability (CR), calculated from standardized factor loadings, showed values between 0.69 and 0.82, considered adequate and, mostly, above the recommended threshold of 0.70 for confirmatory studies [47,51].

As shown in Table 10, the dimensions of speaking in public and interaction with teachers (ω = 0.82), interaction with the opposite sex (ω = 0.76), assertive expression (ω = 0.76), interaction with strangers (ω = 0.79), and acting in public (ω = 0.70) reported satisfactory reliability levels. The ridiculed subscale (ω = 0.69) presents an acceptable value, considering the brevity of the scale (4 items) and the initial nature of the validation process [41,77]. Likewise, all confidence intervals exclude the value zero, which supports the stability of estimates [75].

## 4. Discussion

The results obtained show that the CASO-N24 questionnaire is a robust, multidimensional, and generally invariant tool, useful for assessing social anxiety in Peruvian adolescents; the results of the original theoretical structure of the instrument are replicated, and its applicability is also extended.

This research contributes to empirical validation consistent with the original proposal [26], confirming the robust multidimensional structure with adequate factor loadings and no problematic cross-loadings, even considering the cultural and contextual factors that may influence how adolescents interpret social evaluative situations.

The existence of six dimensions corroborates current contemporary models that consider social anxiety as a heterogeneous and localized construct [34,35]. Recent findings in European and Asian contexts have warned that unidimensional measures tend to underestimate the complexity of this construct, while multidimensional approaches allow for a more precise and reliable assessment of the symptomatic profile [1,2].

The results obtained serve to strengthen the construct validity of the CASO-N24 and make clear that the social situations included in the instrument are valid and representative for Peruvian adolescents. The cumulative explained variance and factor loading indices approximate and even exceed in some cases the results reported in previous validations in other Spanish-speaking countries [29], which constitutes an indication in favor of the structural stability of the model.

The results of confirmatory factor analysis provided suitable fit indices [47,51]. The normed chi-square ratio, both absolute and incremental fit indices, and the low level of standardized residuals indicate that the theoretical model adequately reproduces the observed covariance matrix. These results are relevant, given the ordinal condition of the variables and estimator indices from robust latent variables, which contributes to adequate methodological validity of the results.

Discriminant validity tests based on the HTMT criterion with moderate interfactorial correlations suggest that the evaluated dimensions are interdependent, with distinct facets of social anxiety, consistent with more recent studies that reveal that various social fears have a common basis but are expressed specifically, depending on the type of interaction and evaluative context [14,36].

Complete factorial invariance between the age groups 12–15 and 16–17 years was consolidated. Stability in configural, metric, scalar, and residual models would suggest that the CASO-N24 manages to measure the social anxiety construct comparably across different stages of adolescence. This result finds support in longitudinal research showing that, although social anxiety levels may change with age, the psychological structure of the construct appears to remain stable throughout adolescence [13,19].

From an applied perspective, this measurement equivalence allows for valid comparison of results obtained between adolescents of different age ranges without risk of introducing instrument-related biases. The findings support the age range extension of the CASO-N24, expanding its applicability beyond the age ranges originally proposed by Caballo et al. [26] and strengthening its utility for assessing social anxiety throughout adolescence.

In contrast to age invariance, multigroup analyses by gender found configural and metric invariance but not scalar or residual. That is, the factorial structure and functioning of items are equivalent in males and females, but differences are concentrated in that the intercepts of at least one item are different (item 17) and point to a possible indication of differential item functioning (DIF) by gender, a phenomenon extensively studied in social anxiety assessment.

Adolescent females tend to have higher levels of anxiety associated with social interaction [8,11], which could influence how certain items are interpreted. In this sense, the recent literature shows how fears related to social interaction and interpersonal judgment are associated with gender bias in adolescent age, such as social norms, socialization styles, and even cultural expectations [10,18].

This result shows the sensitivity of the instrument in evaluating social anxiety experiences. Direct comparisons of means between males and females should be performed under a partial invariance approach [6,49,69]. From a developmental and cultural standpoint, the differential functioning of item 17—which pertains to the interaction with the opposite sex dimension—may reflect gender-specific socialization processes that shape how male and female adolescents perceive and respond to heterosocial situations. In many Latin American cultural contexts, gender role expectations impose distinct behavioral norms on adolescent males and females regarding romantic and cross-sex interactions, which may differentially influence the threshold at which anxiety is endorsed in these specific scenarios [33]. Furthermore, pubertal timing and the developmental trajectory of romantic interest differ between genders during adolescence, potentially contributing to divergent interpretations of item content related to opposite-sex interactions [4,6]. These findings carry important practical implications: researchers and clinicians who wish to compare social anxiety scores between males and females using the CASO-N24 should employ partial invariance models that account for item 17, and future instrument refinements could explore alternative item wordings that reduce gender-related differential functioning while preserving content validity.

Evidence of nomological network validity for the CASO-N24 was supported by moderate-high correlations with the ASQ-14, in both males and females. Since the ASQ-14 measures perceived adolescent stress rather than social anxiety per se, these associations are more precisely interpreted as related-construct or nomological evidence—demonstrating that social anxiety relates to a theoretically related but distinct construct—rather than strict convergent validity, which would require correlation with another measure of the same construct. These associations are consistent with theory, which argues that social anxiety constitutes a central core of general psychological distress during adolescence, closely linked to perceived stress, emotional overload, and social demands [15,24].

These correlation magnitudes align with previous research documenting relationships between social anxiety and stress-related variables [19,41], corroborating nomological validity within the expected theoretical network.

Regarding reliability, the omega and alpha coefficients obtained indicate adequate internal consistency for the total scale and all subscales. The omega coefficient used as the main estimator responds to contemporary criteria in psychometrics, used in ordinal scales and factorial models [53,75].

The being ridiculed subscale showed slightly lower coefficients than other dimensions, though still adequate. Prior research has noted similar patterns in subscales with fewer items, without compromising clinical or research utility [77]. The use of structural equation modeling and adequate fit criteria further supports the robustness of the factorial solution obtained [78,79]. The transition from alpha to omega as the preferred reliability estimator reflects current methodological standards in psychometric research [80,81], and is consistent with prior applications of the CASO instrument [82].

Several limitations warrant consideration. The sample included only adolescents from private schools in northern Peru, limiting generalizability to other educational contexts and regions. Future research should include public institutions and rural areas, and employ longitudinal designs to assess temporal stability. Additionally, it should be noted that the CASO-N24 exclusively assesses social anxiety in traditional face-to-face situations and does not capture anxiety experienced in digital or online social interactions. Given that a substantial portion of contemporary adolescents’ social exchanges occurs through social media and other digital platforms, social anxiety may manifest differently in these virtual contexts compared to in-person encounters. Future studies should consider developing or incorporating complementary measures that address social anxiety in digital environments to provide a more comprehensive assessment of this construct in today’s adolescent population.

Future studies should examine predictive validity and sensitivity to change following psychological interventions. Additionally, further exploration of gender-related differential item functioning could facilitate instrument refinement and enhance interpretation of gender differences in social anxiety expression. Furthermore, it must be acknowledged that both the exploratory and confirmatory factor analyses were conducted on the same sample, which may lead to an overestimation of model fit indices. Future studies should ideally employ independent samples or cross-validation strategies (e.g., randomly splitting the sample into calibration and validation subsets) to provide more stringent confirmation of the factorial structure. Moreover, the cross-sectional design of this study precludes the evaluation of temporal stability (test–retest reliability) and predictive validity of the CASO-N24, both of which are essential for establishing the instrument’s utility in longitudinal monitoring and clinical follow-up. Prospective studies incorporating repeated measurements would strengthen the evidence base regarding the instrument’s stability over time. Additionally, the exclusive reliance on self-report measures may introduce response biases, including social desirability and acquiescence tendencies, which are particularly relevant in adolescent populations when assessing socially sensitive constructs such as anxiety. Future research could benefit from incorporating multi-method assessment approaches, such as behavioral observation, peer reports, or physiological indicators, to provide complementary evidence of construct validity.

## 5. Conclusions

This study provides robust evidence for the validity and reliability of the CASO-N24 in Peruvian adolescents aged 12–17 years. Factor analyses confirmed the six-factor structure proposed by Caballo (loadings: 0.48–0.78; variance explained: 47.13%), with excellent model fit (GFI = 0.981; AGFI = 0.976; NFI = 0.971; SRMR = 0.046) and adequate discriminant validity (HTMT criterion).

Complete measurement invariance across age groups (12–15 vs. 16–17 years) supports extending the instrument’s application range. Partial gender invariance was observed, with item 17 showing differential functioning. Convergent validity was confirmed through correlations with ASQ-14 (males: r = 0.622; females: r = 0.604).

Internal consistency was adequate (total scale ω = 0.95; subscales: 0.69–0.82). In sum, the CASO-N24 is a psychometrically sound, culturally pertinent tool for early detection of social anxiety in Peruvian school settings, with direct implications for prevention and mental health intervention programs.

## Figures and Tables

**Table 1:** Configuration matrix of the exploratory factor analysis.

Item	F1	F2	F3	F4	F5	F6
Item 1	0.55					
Item 6	0.80					
Item 8	0.74					
Item 21	0.82					
Item 13		0.65				
Item 17		0.49				
Item 20		0.68				
Item 24		0.83				
Item 2			0.61			
Item 12			0.55			
Item 14			0.70			
Item 19			0.52			
Item 10				0.69		
Item 15				0.65		
Item 18				0.79		
Item 22				0.56		
Item 4					0.73	
Item 7					0.76	
Item 11					0.51	
Item 16					0.79	
Item 3						0.73
Item 5						0.62
Item 9						0.65
Item 23						0.44

Note: Main factor loadings are reported. F1: Speaking in public and interaction with teachers; F2: Interaction with the opposite sex; F3: Being exposed or ridiculed; F4: Assertive expression of annoyance or anger; F5: Interaction with strangers; F6: Acting in public.

**Table 2:** Standardized factor loadings by item in the general model.

Item	F1	F2	F3	F4	F5	F6
Item 1	0.66					
Item 6	0.73					
Item 8	0.78					
Item 21	0.78					
Item 13		0.74				
Item 17		0.48				
Item 20		0.66				
Item 24		0.76				
Item 2			0.61			
Item 12			0.60			
Item 14			0.61			
Item 19			0.59			
Item 10				0.59		
Item 15				0.75		
Item 18				0.62		
Item 22				0.66		
Item 4					0.68	
Item 7					0.70	
Item 11					0.61	
Item 16					0.79	
Item 3						0.68
Item 5						0.58
Item 9						0.56
Item 23						0.61

Notes: F1: Speaking in public and interaction with teachers; F2: Interaction with the opposite sex; F3: Being exposed or ridiculed; F4: Assertive expression of annoyance or anger; F5: Interaction with strangers; F6: Acting in public.

**Table 3:** Evaluation of goodness-of-fit indices of the CFA model.

Index	Abbreviation	Value	Criterion	Met
Chi-squared	CMIN/df	1.550	≤3.0 [47]	Yes
Goodness of Fit Index	GFI	0.981	≥0.95 [47,51]	Yes
Adjusted GFI	AGFI	0.976	≥0.90 [47]	Yes
Normed Fit Index	NFI	0.971	≥0.90 [47]	Yes
Relative Fit Index	RFI	0.966	≥0.90 [51]	Yes
Standardized Root Mean Square Residual	SRMR	0.046	<0.05 [47,51]	Yes
Parsimony Normed Fit Index	PNFI	0.834	≥0.50 [47,51]	Yes

**Table 4:** Correlations between dimensions of the confirmed factorial model.

Factors	Correlation (r)	Factors	Correlation (r)
F4 ↔ F3	0.69	F1 ↔ F4	0.57
F1 ↔ F3	0.68	F2 ↔ F5	0.54
F3 ↔ F6	0.68	F5 ↔ F6	0.52
F1 ↔ F5	0.67	F2 ↔ F4	0.52
F2 ↔ F3	0.67	F1 ↔ F2	0.49
F4 ↔ F5	0.63	F2 ↔ F6	0.47
F3 ↔ F5	0.60	F4 ↔ F6	0.43
F1 ↔ F6	0.58		

Note. F1: Speaking in public and interaction with teachers; F2: Interaction with the opposite sex; F3: Being exposed or ridiculed; F4: Assertive expression of annoyance or anger; F5: Interaction with strangers; F6: Acting in public.

**Table 5:** Multigroup comparison by age group.

Compared Model	CMIN	df	RMR	GFI	AGFI	NFI	RFI	PNFI
Configural	479.45	126	0.051	0.976	0.969	0.963	0.957	0.827
Metric	504.10	108	0.052	0.974	0.969	0.961	0.956	0.856
Scalar	564.34	87	0.055	0.971	0.967	0.956	0.953	0.889
Residual	572.89	63	0.056	0.971	0.968	0.955	0.954	0.930

**Table 6:** Comparison of nested models by age group: 12–15 vs. 16–17 years.

Comparison	Δdf	Δχ^2^	*p*	ΔIFI	ΔTLI	Invariance
Metric vs. Configural	18	24.65	0.002	0.001	0.001	Achieved
Scalar vs. Metric	21	60.24	0.005	0.005	0.003	Achieved
Residual vs. Scalar	24	8.55	0.001	0.001	−0.001	Achieved

**Table 7:** Multigroup comparison between males and females.

Compared Model	CMIN	df	RMR	GFI	AGFI	NFI	RFI	PNFI
Configural	466.12	126	0.047	0.974	0.967	0.959	0.953	0.824
Metric	565.70	108	0.052	0.968	0.962	0.951	0.945	0.847
Scalar	1214.01	87	0.076	0.932	0.921	0.894	0.886	0.831
Residual	1343.91	63	0.083	0.925	0.916	0.883	0.879	0.859

**Table 8:** Comparison of nested models between males and females.

Comparison	Δdf	Δχ^2^	*p*	ΔIFI	ΔTLI	Invariance
Metric vs. Configural	18	99.58	<0.001	0.009	0.008	Achieved
Scalar vs. Metric	21	747.95	<0.001	0.065	0.070	Not achieved

**Table 9:** Standardized factor loadings by item and gender.

Item	Factor	Males	Females
Item 1	F1	0.618	0.698
Item 6	F1	0.655	0.749
Item 8	F1	0.657	0.825
Item 21	F1	0.763	0.766
Item 13	F2	0.632	0.780
Item 17	F2	0.640	0.518
Item 20	F2	0.652	0.621
Item 24	F2	0.788	0.695
Item 2	F4	0.450	0.620
Item12	F4	0.616	0.786
Item 14	F4	0.536	0.642
Item 19	F4	0.595	0.661
Item 10	F3	0.571	0.570
Item 15	F3	0.616	0.573
Item 18	F3	0.554	0.586
Item 22	F3	0.528	0.564
Item 4	F5	0.684	0.670
Item 7	F5	0.646	0.697
Item 11	F5	0.585	0.652
Item 16	F5	0.768	0.796
Item 3	F6	0.718	0.632
Item 5	F6	0.643	0.511
Item 9	F6	0.656	0.654
Item 23	F6	0.492	0.595

Note. F1: Speaking in public and interaction with teachers; F2: Interaction with the opposite sex; F3: Being exposed or ridiculed; F4: Assertive expression of annoyance or anger; F5: Interaction with strangers; F6: Acting in public.

**Table 10:** Reliability coefficients by questionnaire dimensions.

Dimensions	No. Items	α	ω	95% CI Li	Ls
Total test	24	0.90	0.95	0.94	0.96
Speaking in public and interaction with teachers	4	0.82	0.82	0.80	0.84
Interaction with the opposite sex	4	0.75	0.76	0.73	0.79
Being exposed or ridiculed	4	0.69	0.69	0.65	0.72
Assertive expression of annoyance or anger	4	0.76	0.77	0.74	0.80
Interaction with strangers	4	0.79	0.80	0.77	0.82
Acting in public	4	0.70	0.70	0.66	0.74

Notes: α = Cronbach’s Alpha; ω = McDonald’s Omega, recommended for ordinal scales according to Hayes and Coutts [75]. 95% CI = 95% confidence interval of omega coefficients.

## Data Availability

The raw data supporting the conclusions of this article will be made available by the authors on request.
